# Investigation of PZT-5H and PZT-8 type piezoelectric effect on cycling stability on Si-MWCNT containing anode materials

**DOI:** 10.3906/kim-2102-62

**Published:** 2021-10-19

**Authors:** Mehbare DOĞRUSÖZ, M. Taha DEMİRKAN, Rezan DEMİR-ÇAKAN

**Affiliations:** 1 Department of Chemical Engineering, Gebze Technical University, Kocaeli Turkey; 2 Institute of Nanotechnology, Gebze Technical University, Kocaeli Turkey; 3 Department of Material Science and Engineering, Gebze Technical University, Kocaeli Turkey

**Keywords:** Silicon anode, lithium ion battery, piezoelectric additives

## Abstract

Silicon (Si) containing materials cannot be used in commercial lithium ion batteries due to the mechanical stress problem triggered by volume expansion during cycling. The high-volume change causes mechanical instability of Si anode materials during charging/discharging, resulting fast capacity fading. It is thought that piezoelectric materials can be a solution for the volume expansion problem because of their ability to generate electric field when pressure is applied on them. For this purpose, PZT-8 and PZT-5H type piezoelectric materials were mixed with silicon and multiwalled carbon nanotube (MWCNT) to obtain anode composites and tested electrochemically versus lithium metal. The piezoelectiric effect on the electrochemical activity of these anodes is investigated by preparing the anode composite without any piezoelectric material additive (Sample #3). At the end of the 50 charge/discharge cycles, the capacities reached 420 mAh/g, 300 mAh/g and 100 mAh/g for PZT-8-added, PZT-5H-added and no-PZT samples, respectively. These results showed that PZT addition improves capacity performance of Si-MWCNT anodes. Additionally, the obtained anode composites were characterized with X-ray diffraction and scanning electron microscopy.

## 1. Introduction

In recent years, with the developing technology, the use of technological products such as portable electronic devices and electric cars has increased very rapidly [1,2]. According to the Gaines et al., electric car sales in America will reach 40 million by 2050 [3]. Because of high energy density, flexible design opportunities and long cycle lifespans, the lithium ion battery system is more favorable compared with the other battery systems [4,5]. The most common anode material in the current commercial lithium ion battery systems is one of the carbon derivative known as graphite, and its theoretical capacity is 372 mAhg^–1^. This capacity value cannot meet the increasing market demand of the energy storage [6]. Among other potential anode materials, the most promising candidate is silicon (Si) due to its abundancy, high gravimetric capacity (3579 mAhg^–1^), environment friendly nature, relatively low discharge voltage (0.4V) and being well-known material for semiconductor industry [7]. Despite the superior properties of the silicon (Si), ~300% volume expansion occurs during lithiation/delithiation [8]. Thus, the anode particles is gradually pulverized and becomes electrically insulated in the subsequent cycles, additionally unstable solid-electrolyte interface (SEI) is formed [9].

To overcome this problem, a lot of research has been done, and different approaches are being proposed such as nanosizing [10], Si- metal and Si/C composites [11], coating, porous structures [12], thin films [13], Si anode composites with additives [2,8,14]. 

Herein the piezoelectric materials are used as preventing additives for the stress problem emerged during volume expansion as a new approach. Piezoelectric materials have unique properties and are used in many different application such as transducer, sensor, actuators and also special biomedical applications [15]. Addition of PZT (Pb(Zr_x_Ti_1-x_)O_3_ where x is between 0 and 1) materials in Si-C composite anodes can improve the electrochemical performance by these aspects. Firstly, PZT particles in between Si-C composite can act as stress absorption sites that can reduce internal instability caused by the volume changes. Secondly, PZT particles can emerge electrical field affected by the compressive stress during expansion of Si materials that happens during lithiation (reduction of Si particles). The electrical field can help Li ions diffusion deeper and faster in the anode structure. Li ions are normally intercalated and deintercalated through the anode structure during charge and discharge, and this rocking-chair mechanism causes volume expansion and shrinkage. This volume changes can emerge coercive stress onto the Si anode particles. Therefore, this stress can naturally induce local polarization and electric field [8]. Local polarization domains generally tend to emerge in the same direction as they are affected by other nearby domains. In the case of the Li ion diffusion is the same direction with the formed electrical field, Li ions can intercalate faster and in larger amounts compared to the situation without electrical field [9]. By this means, the piezoelectric addition to Si+C anode composite enhance the capacity by improving Li diffusion. Therefore, combination of two effects can bring a better cycling performance and stability. The PZT-5H and PZT-8 type lead containing piezoelectric materials are the best known members of the piezoelectric materials. They have high piezoelectric coefficient (d_33_) as 585 pC/N and 225 pC/N for PZT-5H and PZT-8, respectively. On the other hand, another important parameter is the mechanical quality factors (Q_m_) which are 65 and 1000 for PZT-5H and PZT-8, respectively. The composites were prepared by using PZT-5H and PZT-8 to compare the effect of these parameters on the capacity fading of silicon anode due to the volume expansion. 

## 2. Experimental 

The anode composites were prepared via high-energy ball milling method by mixing nano Si powder (Sigma Aldrich <100 nm particle size), MWCNT (92% purity) and piezoelectric materials with a ratio of 35:35:30, respectively. The particle size was reduced during 90-min mixing in a 10 mL stainless steel jar, while the powder/ball ratio was 1:10. The mixing frequency applied during ball milling was 30 Hz. The electrode slurry was prepared with 10% polyvinylidene fluoride (PVdF), dissolved in N-methyl pyrolidone (NMP). The electrode slurry was coated on copper foil and dried overnight in a vacuum oven. Electrolyte solution was obtained by dissolving the LiPF_6_ salt in EC: DMC (1:1) with 5% FEC addition to stabilize the SEI layer. First two samples coded as Sample #1 and Sample #2 were prepared with PZT-8 and PZT-5H additives, respectively. In order to understand the piezoelectric effect, another sample (Sample #3) was prepared by mixing nano Si powder and MWCNT with a ratio of 50:50, without piezoelectric material addition. Thus, Si/MWCNT ratio was kept the same as of Sample #1 and Sample #2. Whole test cells were prepared inside the argon filled glove box. While cyclic voltammetry tests were conducted by Biologic VMP 3 at 0.1 mV/s scan rate with Swagelock test cell, galvanostatic charge-discharge tests were done by Neware battery tester by assembling the coin test cell inside the argon filled glove box. In order to examine the PZT addition effect, the electrochemical impedance spectra (EIS) was conducted for three samples by using Biologic VMP 3 test station ranging from 0.1 mHz to 1 kHz with 5 mV amplitude. Subsequently the obtained experimental data were fit through EC Lab software Z_fit_.

## 3. Results and discussion

**Figure 1:**
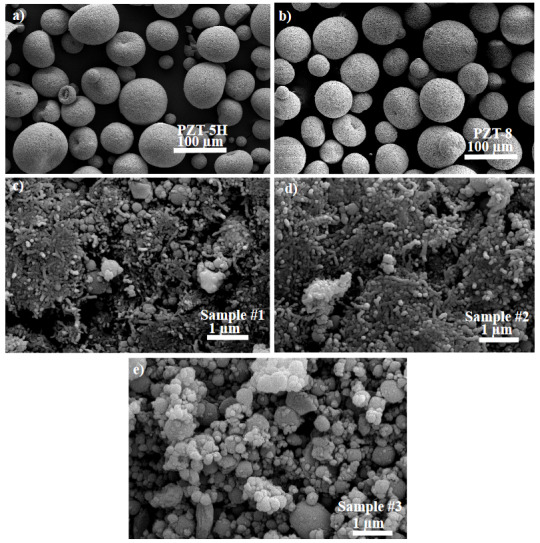
Top view scanning electron microscopy of a) commercial PZT-5H, b) commercial PZT-8, c) Sample #1, d) Sample #2, d) Sample #3.

As-received PZT-5H and PZT-8 samples and ball-milled samples of Si+MWCNT+PZT mixture were characterized by scanning electron microscopy (SEM) in order to see morphological changes during ball milling. PZT- 5H and PZT-8 particles are seen to be spherical and much bigger than the ones in the milled sample as shown in Figures 1a and 1b, respectively. The particle size was reduced to submicron scale after ball milling as seen in Figures 1c–1e for Sample #1, Sample #2 and Sample #3, respectively. The multiwalled carbon nanotubes are seen to mixed homoegenously with PZT and Si particles in which is defined in Figures 1c and 1d. Sample #3 was prepared to confirm the PZT effect without MWCNT addition and in Figure 1e there is submicron sized particles with homogenous mixing.

**Figure 2:**
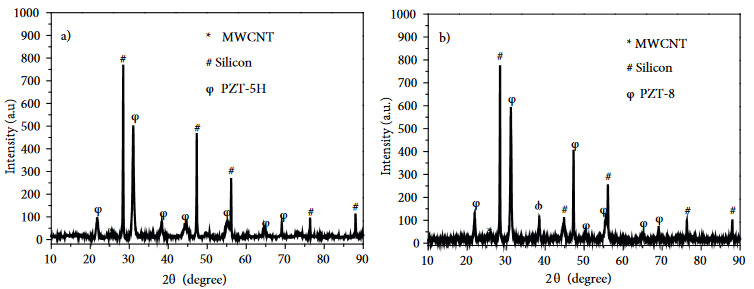
(a) X-Ray diffaraction pattern of milled Sample #1. (b) X-ray diffaraction pattern of milled Sample #2.

X-ray diffraction pattern of the ball-milled samples (Figure 2a) showed only one peak of MWCNT at 2*θ* = 25, two main peaks for Si at 2*θ* = 28 and 2*θ* = 47, and three main peaks for PZT-5H at 2*θ* = 31, 2*θ* = 38 and 2*θ* = 55. It should be noted that the XRD data was taken after ball milling and before electrochemical tests, thus it confirms that the crystalline structure of PZT-5H is still intact after the milling even though the particle size is reduced. X-ray diffraction pattern (Figure 2b) showed only one peak of MWCNT at 2*θ* = 25; two main peaks for Si at 2*θ* = 28 and 2*θ* = 47, and three main peaks for PZT-8 at 2*θ* = 31, 2*θ* =38 and 2*θ* = 55. Similar comment for the XRD plot of PZT-5H can be also made for PZT-8 as the crystalline structure of PZT-8 is still intact after ball milling according to the XRD data.

**Figure 3:**
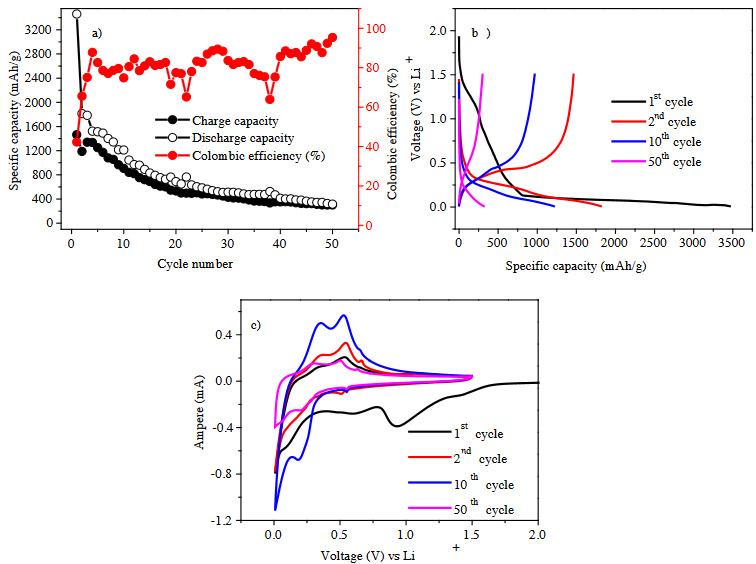
(a) Cycling performance, (b) charge-discharge profiles, (c) cyclic voltammogram of Sample #1. Note: Electrodes were tested at C/5 rate in the voltage range of 0.01–1.50 V vs. Li+/Li.

Figure 3a depicts the charge/discharge profiles of Sample #1 that was prepared by PZT-5H type piezoelectric. Charge/discharge profiles are used to investigate the effect on capacity fading of silicon anode in lithium ion battery system. The columbic efficiency line is fluctuated between 80% and 100% while the specific capacity reduces to approximately 300 mAh/g after 50 chare-discharge cycles. This high capacity fading is attributed to volume expansion of silicon anode where the addition of PZT-5H type piezoelectric was not found to be sufficient to suppress the volume expansion. The similar fading also is seen in the voltage profiles of Sample #1 (Figure 3b), as gradual capacity fading is apparent after the second charge-discharge cycle. The cyclic voltammogram (CV) (Figure 3c) also supports the capacity fading, because of the reduced peaks intensities in each cycle. 

**Figure 4:**
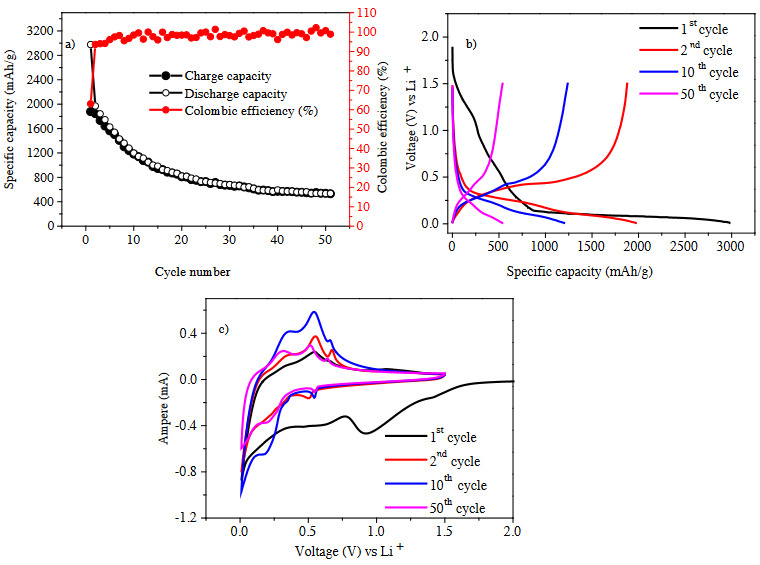
(a) Cycling performance, (b) charge-discharge profiles, (c) cyclic voltammogram of Sample #2. Note: Electrodes were tested at C/5 rate in the voltage range of 0.01–1.50 V vs. Li+/Li.

The cyling performance of Sample #2 is displayed in Figure 4a. The colombic efficiency line is more stable than the Sample #1 as the colombic efficiency trends around >97%. After 50 charge-discharge cycles, the capacity value reaches approximately 500 mAh/g. Addition of PZT-8 type piezoelectric material seems to be more successful to suppress the mechanical stress caused by volume expansion of Si than the PZT-5H type material. The CV (Figure 4c) confirms the capacity fading as the peaks were shifted during cycling. In CV graphs in Figures 3c and 4c, the first scans only have one reduction peak at ~0.7 V, which is attributed to the SEI layer formation caused by Li and electrolyte interaction. Additionally in the second scan, the peaks at 0.5 V, 0.2 V and 0.01 V correspond to alloying step of Li^+^ ion [16]. Lead based piezoelectric materials contain PbO phase within its crystalline structure. Besides the Si anode oxidation reduction peaks, the PbO phase can have four anodic peaks at 0.6 V, 0.45 V, 0.37 V, 0.29 V corresponding to formation of LiPb, Li_3_Pb, Li_3.2_Pb, Li_4.5_Pb phases respectively, whereas three cathodic peaks of 0.53 V, 0.4 V and 0.27 V referring to PbO phase conversion [17,18]. CV plateau confirm the PbO and Si anode oxidation reduction peaks interference at the near voltage point. After the 50th charge-discharge cycle, PbO phase oxidation peak at 0.6 V is not visible meaning that PbO is contributing to the capacity to some extend until the 50th cycle. However, improved capacity performance of the anode is more likely due to the piezoelectric effect of PZT materials. Resultantly the capacity fading could be explained by two factors: (1) piezoelectric effect loss due to the loss of polarization or (2) the side reactions. The side reactions can be attributed to PZT particle decomposition during cycling, and thus to the loss of PZT particles and decreased piezoelectric effect.

**Figure 5:**
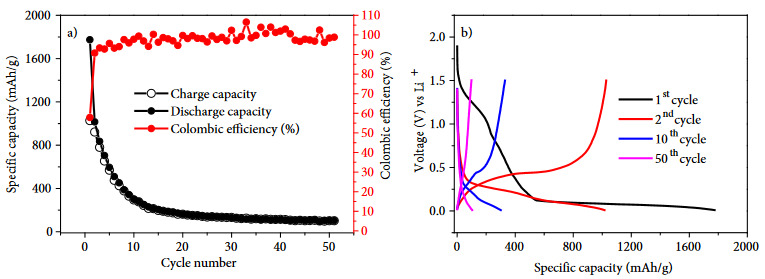
(a) Cycling performance, (b) charge-discharge profiles of Sample #3. Note: Electrodes were tested at C/5 rate in the voltage range of 0.01–1.50 V vs. Li+/Li.

In Figure 5 the charge-discharge profiles and cyling performance of Sample #3 –which has no addition of PZT materials– was shown. The capacity value reaches 100 mAh/g after 50 charge-discharge cycles later. The capacity fading ocurred more rapidly comparing with the PZT-added samples verifiying the piezoelectric effect on the electrochemical performance. 

**Figure 6:**
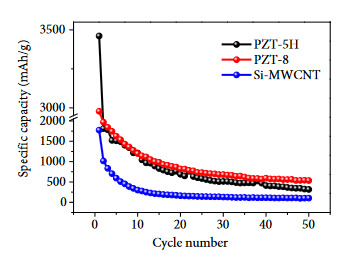
Discharge comparison of Si-C composites.

In Figure 6, the discharge capacities comparison of the PZT-added and nonadded composites were summarized. Both PZT-5H-added (Sample #1) and PZT-8-added (Sample #2) Si/MWCNT composite samples showed better performance compared to the non-PZT sample (Sample #3). This confirms that addition of PZT materials have a positive effect on electrochemical performance. Considering the fact that there is an ~200%–300% improvement in the capacity when there is only 30% addition of PZT materials, it can be inferred that this capacity increase is emerged from piezoelectric effect and not from the reactions of PbO. Moreover, PZT-8 added sample gave better and more stable discharge capacity than PZT-8-added sample. While PZT-8-added sample can reach approximately 420 mAh/g, PZT-5H-added sample delivers approximately 300 mAh/g discharge capacity value at the 50th cycle. 

**Figure 7:**
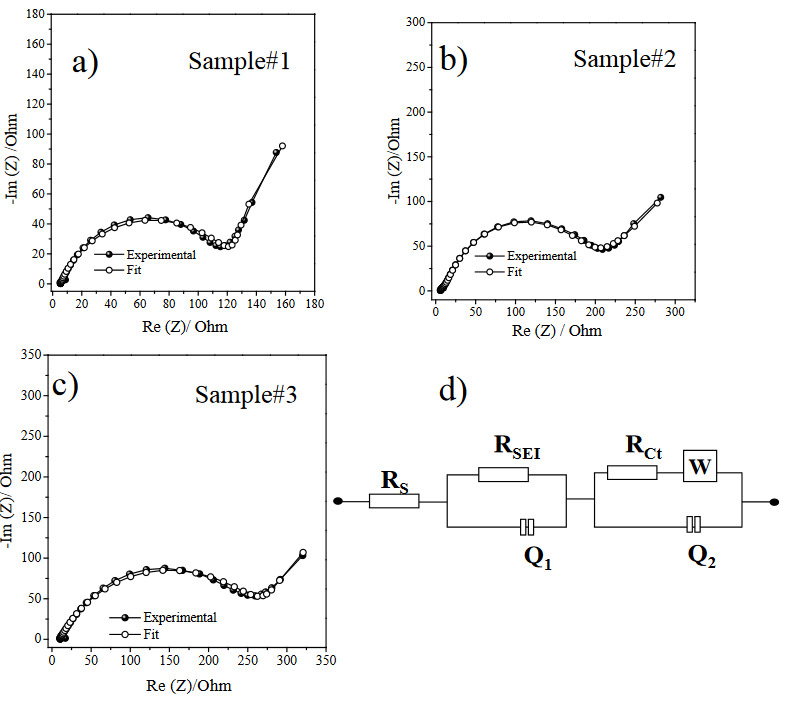
Electrochemical impedance spectrum of experimental and fit data of a) Sample #1, b) Sample #2, c) Sample #3, d) equivalent circuit.

Electrochemical impedance spectra of three samples is demonstrated in Figure 7. The equivalent circuit model has electrolyte resistance (R_e_), charge transfer resistance (R_ct_), electrode-electrolyte interface resistance (R_SEI_). The W symbolized Warburg element in which is described the Li ion diffusion related with the inclined line part of Nyquist plot [19]. Q_1_ and Q_2_ represent the constant phase element related with the electrode-electrolyte interface resistance and charge transfer resistance respectively [20]. The depressed semicircle corresponds to high and medium frequency region therefore R*_SEI_* and R*_ct_*, respectively. R*_total _* is the total interfacial resistance and sum of R*_SEI_* and R*_ct _* [21].** According to the experimental data fitting results, R*_total _* are 414.9 Ω, 178.4 Ω and 666.8 Ω for Sample #1, Sample #2 and Sample #3, respectively. Consequently the Sample #2 has the lower interfacial resistance in which the lowest capacity fading occurs. Comparing three samples, interfacial resistance of PZT added samples are far less than the sample without PZT addition. That verifies the effect of PZT addition on the electrochemical performance.

When the piezoelectric properties are considered, PZT-5H and PZT-8 are defined as soft and hard piezoelectric materials, respectively, meaning the piezoelectric materials are polarized easily or hardly. It can be assumed that PZT-8 –which could be polarized under difficult conditions– creates an electric field only after the stress accumulated in Si reaches a certain amount. If this is the case, it can be presumed that PZT-8 helps Li-ions to intercalate at a time when the lithiation become difficult because of the expansion during charging. While piezoelectric coefficient of the PZT-5H higher than the PZT-8, the mechanical quality factor of PZT-8 is higher (1000) than PZT-5H, providing lower mechanical loss during cycling. Moreover, the stress accumulated in the Si anode can be 300 MPa during Li_x_S formation [22]. PZT-5H was shown to preserve its piezoelectricity properties under 150 MPa stress cycle, while this value is 280 MPa for PZT-8, meaning that PZT-8 is able to accommodate more stress without losing its piezoelectric properties for longer charge-discharge cycles[23]. Although this shows a reasonable relationship between piezoelectric properties and electrochemical performance, further experiments are needed to confirm it.

## 4. Conclusion

Piezoelectric materials are special group that can generate the electricity when the external pressure applied. This property can provide a novel way for the suppression of the internal stress which is caused by volume expansion of the Si anode while also their electric field effect can be used to improve Li intercalation ability. The resulting Si/MWCNT composites were tested against Li metal as anodes and the capacity value reached to 420 mAhg^–1^, 300 mAhg^–1^, 100 mAhg^–1^ for PZT -8, PZT-5 added and nonadded PZT samples, respectively. In general, the results show that PZT addition to anode materials improves electrochemical performance due to piezoelectric effect although some contribution of PZT to the capacity as an active material was also observed. However, 300% capacity increase from 30% PZT addition shows that the contribution comes mostly from piezoelectric effect. In order to further investigate and confirm the piezoelectric effects on electrochemical performances of anode materials, piezoelectric materials which have different dielectric constant and quality factor values such as PMN based materials (d_33 = _~700 pC/N) can be tested in future.
